# Spontaneous mutation in the *COL6A2* gene causing Ullrich congenital muscular dystrophy type 1 in a Chinese child: A case report

**DOI:** 10.1097/MD.0000000000036398

**Published:** 2023-12-08

**Authors:** Jiayi Li, Shuangzhu Lin, Qiong Wu, Jinhua Feng, Qiandui Chen, Kai Jiang

**Affiliations:** a Changchun University of Chinese Medicine, Changchun, China; b Diagnosis and Treatment Center for Children, Affiliated Hospital of Changchun University of Chinese Medicine, Changchun, China.

**Keywords:** children, COL6A2 gene, Ullrich congenital muscular dystrophy type 1

## Abstract

**Rationale:**

Mutations in the gene encoding type VI collagen cause Bethlem myopathy (MIM 158810) and Ullrich congenital muscular dystrophy (MIM 254090); 2 diseases previously recognized as completely independent, and have been increasingly recognized. However, collagen-related myopathy caused by intron variation in the *COL6* gene is rarely reported in China. Ullrich congenital muscular dystrophy is an autosomal recessive disorder that leads to severe muscle weakness with early onset. Thus, children may never walk independently, with proximal joint contractures and significant hyperelastic distal joints, and have early respiratory failure. Therefore, timely diagnosis and treatment are important. We report a spontaneous mutation in the *COL6A2* gene causing Ullrich congenital muscular dystrophy type 1 in a pediatric patient.

**Patient concerns:**

A boy aged 4 years was unable to walk independently, could sit alone for a short time, and his motor development was delayed and had regressed after 1 year of age. He had a high palatal arch and a through palm with localized transverse lines running laterally from the palm. Electromyography showed an impaired neurogenic source, and whole-exon gene sequencing revealed a spontaneous heterozygous mutation in the COL6A2 gene (c.955-2A>G), which was determined to be a pathogenic mutation according to the American Guidelines of the College of Medical Genetics.

**Diagnoses:**

This child has a delayed motor development, high osprey arch and a through palm with localized transverse lines running laterally from the palm, and regression of motor development after the age of 1 year. Whole exon examination showed spontaneous mutation of the *COL6A2* gene; thus, the child was diagnosed with UCMD type 1.

**Interventions:**

At present, there is no special treatment for this disease, and treatment is mainly symptomatic and supportive. The child underwent home massage, rehabilitation training, oral folic acid tablets, vitamins and coenzyme Q10.

**Outcomes:**

During the subsequent follow-up period, the patient can now sit alone for a short period of time.

**Lessons:**

We report a case of spontaneous mutation in the COL6A2 gene causing Ullrich congenital muscular dystrophy type 1 in a pediatric patient, expanding the phenotypic spectrum of the disease and enriching the human gene pool.

## 1. Introduction

Ullrich muscular dystrophy is a congenital myopathy. The pathogenesis of this disease is related to a collagen synthesis disorder. The loss of association between the stroma and the basal layer may be a novel molecular pathological mechanism underlying muscular dystrophy. A key factor in the development of muscular dystrophy is the selective lack of collagen VI in the endomysium. Collagen VI is a ubiquitous extracellular matrix protein and is particularly enriched in the endomysium of skeletal muscle. It binds tightly with the basement membrane surrounding muscle cells to form a microfibrillary network, and is able to interact with several other matrix components. Collagen VI consists of 3 different alpha chains encoded by separate genes named COL6A1, COL6A2, and COL6A3. Mutations in the collagen VI gene causes 2 skeletal muscle disorders, namely, Bethlem myopathy and Ullrich congenital muscular dystrophy (UCMD) type 1. The *COL6* gene (*COL6A1, COL6A2*, and *COL6A3*) variants cause defects in collagen function, and the main clinical manifestations are motor retardation, limb muscle weakness (proximal greater than distal), high palatal arch, slender limbs, proximal joint contracture, and distal joint laxity. Generally, in the neonatal period or early infancy^[1,2]^ malnutrition implies that muscle histopathology shows features of necrosis and regeneration followed by adipose and fibrotic tissue genesis. Muscle biopsy may also show other nonspecific signs, such as fibrous atrophy, hypertrophy, and increased inflammation. We report a spontaneous mutation in the *COL6A2* gene causing UCMD type 1 in a pediatric patient.

## 2. Case presentation

A 4-year-old boy, born in October 2018, presented to the clinic on 20 July 2022 due to motor retardation with regression for >3 years.

### 2.1. History of present illness

When the child was age 4 months, the family felt that his motor development was slower than that of other children of the same age, and he could not pull himself up or turn over. Therefore, they attended the local hospital. The child was diagnosed with backward development, and underwent rehabilitation training. When the child was 6 months old, head magnetic resonance imaging (MRI) and chromosome copy number examination showed no abnormalities, and rehabilitation training was continued. The child visited the hospital again in November and was admitted for treatment. Genetic myopathy was not excluded. Subsequently, he attended several hospitals due to the existence of muscle disease, and continued rehabilitation training. At the age of 2 years, the child was again admitted to the local hospital for treatment.

### 2.2. History of past illness

The child weighed 3.4 kg at birth, turned over at 4 months, looked up at 5 months, was unstable at 6 months, sat alone at 8 months, crawled at 12 months, and showed developmental regression at 1 year. He received rehabilitation training, and by the age of 4 years, he still could not walk alone and could only sit alone for a short time. The child had no previous history of hepatitis, tuberculosis, drug or food allergies, and no surgical trauma. Vaccinations were carried out as planned.

### 2.3. Personal and family history

The patient (G2P2) had no history of asphyxiation, hypoxia, umbilical cord wrapped around his neck, or dystocia. The mother was healthy during pregnancy, denied taking medication, and had no history of poisoning. The father was in good health. There was no family history of children with language and intellectual developmental disorders.

### 2.4. Physical examination

The general status and mental state of the child were assessed. No rash on the entire body or coffee spots/depigmentation on the skin were noted, his face was pale, with a low nose bridge, slightly wide distance between the eyes, a high palatal arch and a through palm with localized transverse lines running laterally from the palm, no swollen lymph nodes in the neck, thick breathing sounds in both lungs, and dry and wet rales were not heard. Strong heart sounds, normal heart rate, normal rhythm, soft abdomen, no tenderness, no rebound pain and muscle tension, no ribs palpated under the liver and spleen, and normal bowel sounds were detected. Neurological examination revealed the proximal muscle strength of the upper limbs was level III, the distal muscle strength was level IV, and the lower limb muscle strength was III+; weakened muscle tension in all limbs; and he did not cooperate with the ataxia test. The biceps reflex, triceps reflex, radial F reflex, knee tendon reflex and Achilles tendon reflex were not elicited. He was negative for ankylosis, Brudzinski’s sign and Kernig’s sign, and had no abnormalities in the remainder of the neurological examination.

### 2.5. Laboratory examinations

On January 31, 2019, no abnormalities in blood and urine screening for inherited metabolic diseases were observed. On April 9, 2019, detection of chromosomal copy number variation showed no pathogenic copy number variation. On September 25, 2019, blood lactate, blood ammonia, routine 5-item thyroid function test, parathyroid hormone, liver function, myocardial enzymes, ions, renal function, iron metabolism, homocysteine, routine blood coagulation, immunity, and 25-hydroxyvitamin D3 were normal. No abnormalities were found on electrocardiography. Electromyography was performed to determine sensory nerve conduction velocity and the results were as follows: ulnar nerve sensory left wrist V-wrist conduction velocity: 39.1 m/s; sensory left finger-wrist conduction velocity of the median nerve: 40.4 m/s; conduction velocity of the middle-external malleolus of the left lower leg sural nerve: 44.6 m/s; motor nerve conduction velocity, F wave: ulnar nerve movement left wrist-small finger abductor elbow-wrist conduction velocity: 44.6 m/s; median nerve movement left wrist - short thumb adductor elbow - wrist conduction velocity: 42.8m/s; tibial nerve movement left ankle-squat abductor popliteal-ankle conduction velocity: 37.5 m/s; small head-ankle conduction velocity of the left ankle-short abductor of common peroneal nerve movement: 47.9 m/s.

### 2.6. Imaging examinations

On April 9, 2019, no abnormalities were observed on head MRI. On September 25, 2019, no abnormalities on abdominal color ultrasound and double hip positive digital radiography were found. MRI showed that both thigh muscles were thin, and a small amount of effusion was seen in both hips. On April 8, 2020, cardiac color ultrasound revealed atrial septal defect (secondary hole type) (the gap was 2.7 mm long).Considering that the immunohistochemical detection of muscle tissue collagen is invasive, the parents refused this examination. Immunohistochemical testing of muscle tissue collagen can be performed to further clarify the diagnosis and guide treatment.

### 2.7. Results of genetic analysis

**Figure 1.**
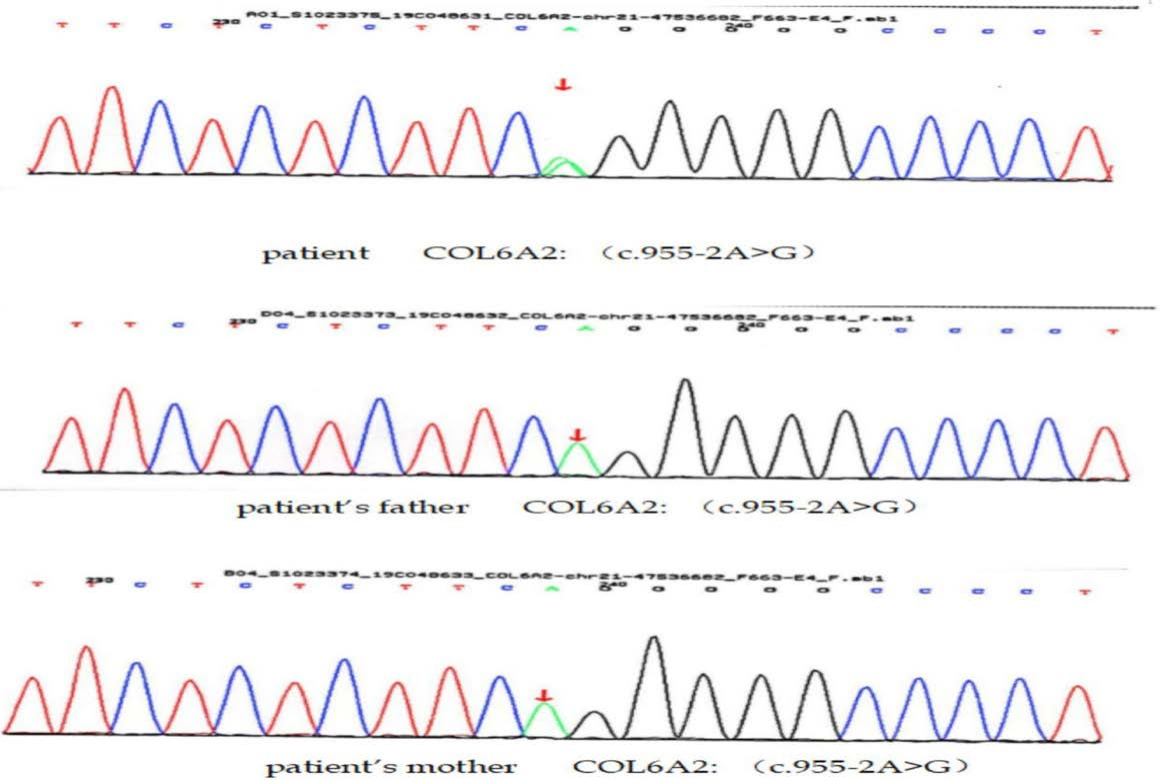
Genogram of COL6A2: (c.955-2A > G).

After obtaining informed consent from the parents, peripheral blood was collected from the patient and his parents for whole-exome gene sequencing, which revealed a spontaneous mutation in the *COL6A2* gene (c.955-2A > G) (Fig. 1) at chromosomal position chr 21:47536628, transcript exon NM-058174; exon 10, nucleotide amino acids c.955-2A > G (splicing). For the splicing variant, disease/phenotype was as follows: UCMD type 1; Bethlem myopathy type 1; and autosomal recessive muscle sclerosis. The bioinformatics protein function prediction software SIFT, PolyPhen-2, MutationTaster, GERP++, and REVEL were predicted to be unknown, unknown, deleterious, deleterious, and unknown, respectively. According to the guidelines of the American College of Medical Genetics, the pathogenic variant was classified as PVS 1, PM2-Supporting, PM3-Strong, and was clinically diagnosed as UCMD type 1.

## 3. Discussion

The final diagnosis of UCMD type 1 was made by genetic analysis, and *COL6A2* showed a variety of phenotypes ranging from milder Bethlem myopathy to more severe UCMD. Before and during the era of NGS, several studies have shown the genetic spectrum of type VI collagen-associated malnutrition. The most common is glycine substitution in the triple helical domain, with other missense variants, nonsense variants, splice variants that cause exon skipping, small in-frame deletions/insertions, and small deletions/insertions leading to premature stop codons. Large genomic deletions spanning multiple exons are rare.^[3–5]^

Our patient was 4 years old and presented with motor retardation with regression for >3 years. The combination of developmental delay with regression and his clinical presentation attracted our attention. We strongly suspected that this child had a muscle-related disease or a genetic metabolic disease. Electromyography suggested motor nerve conduction velocity block of the median nerve in both upper limbs; and neurogenic damage in all limbs. The child’s chromosomal copy results were normal, and the whole exon test was performed for further diagnosis and treatment, and the results revealed spontaneous mutation of the *COL6A2* gene. A pathogenic variant was considered according to the American College of Medical Genetics guidelines. This led to confirmation of the diagnosis of UCMD type 1.

The main clinical manifestations and genetic test results in this patient were in line with UCMD type 1. Unfortunately, we could not perform further immunohistochemical testing of muscle tissue collagen. During the process of clinical diagnosis and treatment, we considered the possibility of other muscular diseases,^[6]^ including Bethlem myopathy and limb muscular dystrophy,^[7]^ but the clinical manifestations and laboratory results excluded both diseases. The clinical features in this child were more severe than those of Bethlem myopathy, which is characterized by slow progressive midaxial and proximal muscle weakness with flexural finger contractures. The disease exhibits intrafamilial variability with variable onset (from prenatal onset to middle adulthood onset), and is usually mild but can be slowly progressive, and some affected individuals >50 years old require assistance with outdoor activities. The mode of inheritance is autosomal dominant and the mutations can affect any of the three *COL6* genes. It has been suggested that at least some cases of Ullrich’s disease are caused by recessive mutations in *COL6A2*.^[8]^ Collagen VI expression is normal or slightly reduced in the endomysium in most patients. Limb girdle muscular dystrophy is a genetic disorder that mainly affects skeletal muscle, leading to progressive muscle atrophy, mainly muscle fiber loss and proximal muscle weakness. However, evidence of degenerative changes must be accompanied by an increase in serum creatine kinase activity.

## 4. Conclusion

We report the case of a spontaneous mutation in the *COL6A2* gene causing UCMD type 1 in a pediatric patient, expanding the phenotypic spectrum of the disease and enriching the human gene pool. The patient’s condition will continue to be monitored and he can undergo immunohistochemical testing of muscle tissue collagen when conditions permit.

## Acknowledgments

We thank the patient and their family for their participation and support in this study. We thank Liu Yushu National Famous Elderly Chinese Medicine Experts Inheritance Workshop for financial support of this article.

## Author contributions

**Data curation:** Qiong Wu, Jinhua Feng, Qiandui Chen.

**Writing – original draft:** Jiayi Li.

**Writing – review & editing:** Shuangzhu Lin, Kai Jiang.
